# Adapting Pressurized Sample Infusion ESI‐MS for Real‐Time Monitoring of Heterogeneous Photoelectrocatalytic Reactions

**DOI:** 10.1002/jms.70129

**Published:** 2026-11-01

**Authors:** Bobby A. Ryane, Bragi Zhang, Maria E. Muraro, Hannah A. Trieu, Heather L. Buckley, J. Scott McIndoe

**Affiliations:** ^1^ Department of Chemistry University of Victoria Victoria British Columbia Canada; ^2^ Centre for Advanced Materials and Related Technology (CAMTEC) University of Victoria Victoria British Columbia Canada; ^3^ Department of Civil Engineering University of Victoria Victoria British Columbia Canada; ^4^ Institute for Integrated Energy Systems (IESVic) University of Victoria Victoria British Columbia Canada

## Abstract

Pressurized sample infusion (PSI) enables real‐time electrospray ionization mass spectrometric (ESI‐MS) monitoring of reactions through continuous delivery of solution from a pressurized vessel. Existing PSI configurations, however, are not well suited to systems involving heterogeneous catalysts with strict geometric demands, such as TiO_2_ nanotube plates. Here, we describe a modified PSI configuration in which a standard media bottle fitted with a silicone septum replaces a multineck Schlenk flask. This design accommodates plate‐based catalysts that cannot be easily used in conventional PSI reactors (in this specific case, TiO_2_ nanotube arrays), enables controlled pressurization via needle‐based gas injection, and permits insertion of capillary tubing for continuous sample infusion to the mass spectrometer. Rhodamine B was used as a model compound to monitor photocatalytic (PC) and photoelectrocatalytic (PEC) degradation under different reaction conditions. The approach enables straightforward real‐time mass spectrometric monitoring of PEC and PC systems employing heterogeneous catalysts.

## 1 Introduction

Pressurized sample infusion electrospray ionization mass spectrometry (PSI‐ESI‐MS) enables real‐time monitoring of reactions by continuously delivering solution from a pressurized vessel directly to a mass spectrometer. Originally developed to study air‐ and moisture‐sensitive organometallic reactions [1], the technique was later extended to a variety of systems [2] and pressure sources [3]. More broadly, developments in on‐line mass spectrometric monitoring have enabled the detection of reaction transformation products using alternative ionization strategies, such as inductive electrospray ionization sources [4], and multichannel peristaltic pump systems coupled to a custom electrospray nozzle and a linear ion trap mass spectrometer for real‐time reaction monitoring [5]. PSI exploits the high sensitivity and high resolution of ESI‐MS to follow reaction progress, transformation products, and kinetics with minimal delay between sampling and analysis. A pressure gradient forces the reaction mixture through PEEK tubing into the ESI source, allowing continuous, on‐line observation of reaction chemistry. Since its introduction, PSI‐ESI‐MS has been applied to a wide range of systems to uncover mechanistic insights into transient species that are often inaccessible by conventional off‐line methods [6, 7, 8, 9, 10]. Despite its utility, the technique remains limited to homogeneous reaction mixtures, and reactions that take place at solid surfaces or interfaces have not been monitored using conventional PSI configurations.

Photoelectrocatalytic (PEC) and photocatalytic (PC) systems are used in applications including pollutant degradation, chemical synthesis, and solar fuel generation such as hydrogen production from water splitting [11, 12, 13, 14]. A typical reaction setup may involve electrical contacts and a light source, with sample aliquots periodically withdrawn at defined time intervals and analyzed using UV‐Vis spectroscopy, high performance liquid chromatography (HPLC), mass spectrometry (MS), or NMR spectroscopy [15, 16, 17, 18, 19]. Real‐time UV‐Vis spectroscopy has also been employed for continuous monitoring of homogenous systems [20]; however, it generally cannot distinguish between parent compounds and transformation products that possess similar optical properties. Discrete, off‐line analyses provide fewer data points compared to continuous monitoring approaches such as PSI‐ESI‐MS, which can provide time‐resolved information on the scale of seconds to minutes. As a result, PSI‐ESI‐MS can facilitate the detection of short‐lived transformation products and reactive intermediates that may be missed using conventional off‐line sampling methods [21]. Furthermore, delays between sample collections and analysis can permit continued reaction, degradation, or adsorption processes to occur, potentially altering the measured composition of the sample. Consequently, real‐time monitoring of PEC or PC reactions can provide mechanistic insights into reaction pathways and transformation product evolution [22]. An additional advantage of PSI‐ESI‐MS is the ability to differentiate true transformation products from persistent background species. For example, a signal observed at *m/z* 282.3, commonly associated with plastic‐derived contaminants (e.g., protonated oleamide) [23], may appear to increase as the parent ion signal decreases (despite remaining relatively constant in absolute intensity), an effect that can arise from normalization or relative scaling of the data. In off‐line, discrete sampling, this artifact may lead to misassignment as a reaction intermediate. In contrast, PSI‐ESI‐MS enables continuous monitoring of extracted ion traces, allowing species with flat chromatograms to be distinguished from ions that exhibit an observable formation and possible subsequent depletion, behavior consistent with a reaction intermediate (Figure S1). On‐line monitoring techniques with high time resolution enable a deeper understanding of reaction mechanisms, transient transformation products, and catalyst design.

Monitoring heterogeneous catalytic systems, particularly those involving planar or plate‐based catalysts such as BEEP [15], is more challenging than homogeneous systems. Difficulties arise because conventional PSI configurations are not well suited to reactors containing large solid catalysts or geometries that require larger reaction vessels. Larger standard Schlenk flasks commonly used for PSI experiments typically have neck sizes of 24/40 or 24/29, corresponding to minimum internal diameters of 20–21 mm, which restrict the size and geometry of catalysts that can be inserted while maintaining proper reactor configuration. Although a custom‐made Schlenk flask with widened necks (which require custom fitted rubber septa) or elongated bodies could accommodate larger catalysts and increase the catalyst to liquid ratio, custom glassware is costly or requires access to specialized glassblowing facilities. As a result, alternative reactor configurations that enable PSI‐ESI‐MS monitoring of plate‐based heterogeneous catalytic systems are needed.

In this work, we demonstrate a simplified PSI‐ESI‐MS configuration incorporating a media bottle, septum, and needle‐injected gas that allows real‐time monitoring of heterogeneous catalytic systems while reducing both experimental cost and complexity. Rhodamine B was chosen to validate the PSI‐ESI‐MS setup because it has been extensively used as a model dye for PC and PEC systems [24, 25]. Photocatalytic degradation of dyes such as Rhodamine B is commonly monitored using UV‐Vis spectroscopy, which may not distinguish between decolorization and true mineralization or transformation pathways [26, 27]. Previous studies have employed ESI‐MS and LC‐MS to identify Rhodamine B transformation products and elucidate degradation pathways, highlighting the additional molecular‐level information provided by mass spectrometry [28, 29, 30, 31]. Mass spectrometry can provide detailed information on transformation products. However, when used off‐line or focused primarily on parent ion decay, changes in reaction pathways or formation of transient products may be overlooked [32]. PSI‐ESI‐MS was used to investigate reaction pathways and halogenation effects in real‐time during PEC and PC degradation of Rhodamine B in aqueous solution.

## 2 Experimental

### 2.1 PSI Setup

TiO_2_ nanotube array electrodes (38 × 64 mm) were prepared by electrochemical anodization of titanium plates followed by thermal annealing, as previously described [15]. A 100‐mL GL45 media bottle was sealed with a polypropylene (PP) screw cap modified with a 35‐mm hole fitted with a 43‐mm diameter silicone rubber septum. A TiO_2_ plate catalyst (38 × 64 mm) with two titanium leads spot‐welded to the upper edge served as the working electrode. A titanium mesh counter electrode, with two titanium leads woven through the mesh, was positioned opposite the plate. Dual leads were used on each electrode to prevent rotational motion within the vessel. All leads were passed through the septum. The mesh electrode allowed UV light to be positioned directly in front of the mesh permitting light to pass through and illuminate the TiO_2_ nanotube array plate. For consistent degradation profiles, the catalyst and counter‐electrodes were held in place with either electrical tape around the leads or a 3D printed plate holder inserted into the media bottle. Additional photographs detailing the reactor construction, electrode mounting, gas delivery, and sample transfer connections are provided in the Supporting Information (Figure S2). Although silicone septa were used throughout this work, other chemically compatible septa may also be employed.

Pressurization was achieved by introducing oxygen (grade 2.0, 99.0% purity) through a 21‐gauge stainless steel needle and 0.2‐μm syringe filter connected to VWR vacuum rubber tubing (3/16″ ID), maintaining an internal pressure of 5 psi. Sample transfer was accomplished using PEEK tubing (1/16″ OD, 0.005″ ID) inserted through the septum extending into the solution.

A syringe pump (New Era, Model Number NE‐1000) delivered acetonitrile from a 10‐mL syringe at 20.00 μL min^−1^ to a static mixer (Idex High‐Pressure MicroStatic Mixing Tee assembly) through PEEK (0.005″ ID), which was connected to the PEEK transfer line from the pressurized reaction vessel. The mixed stream was continuously infused into the electrospray ionization source using PEEK tubing (0.010″ ID) for real‐time mass spectrometric analysis. A schematic of the full setup is shown in Figure 1.

**FIGURE 1:**
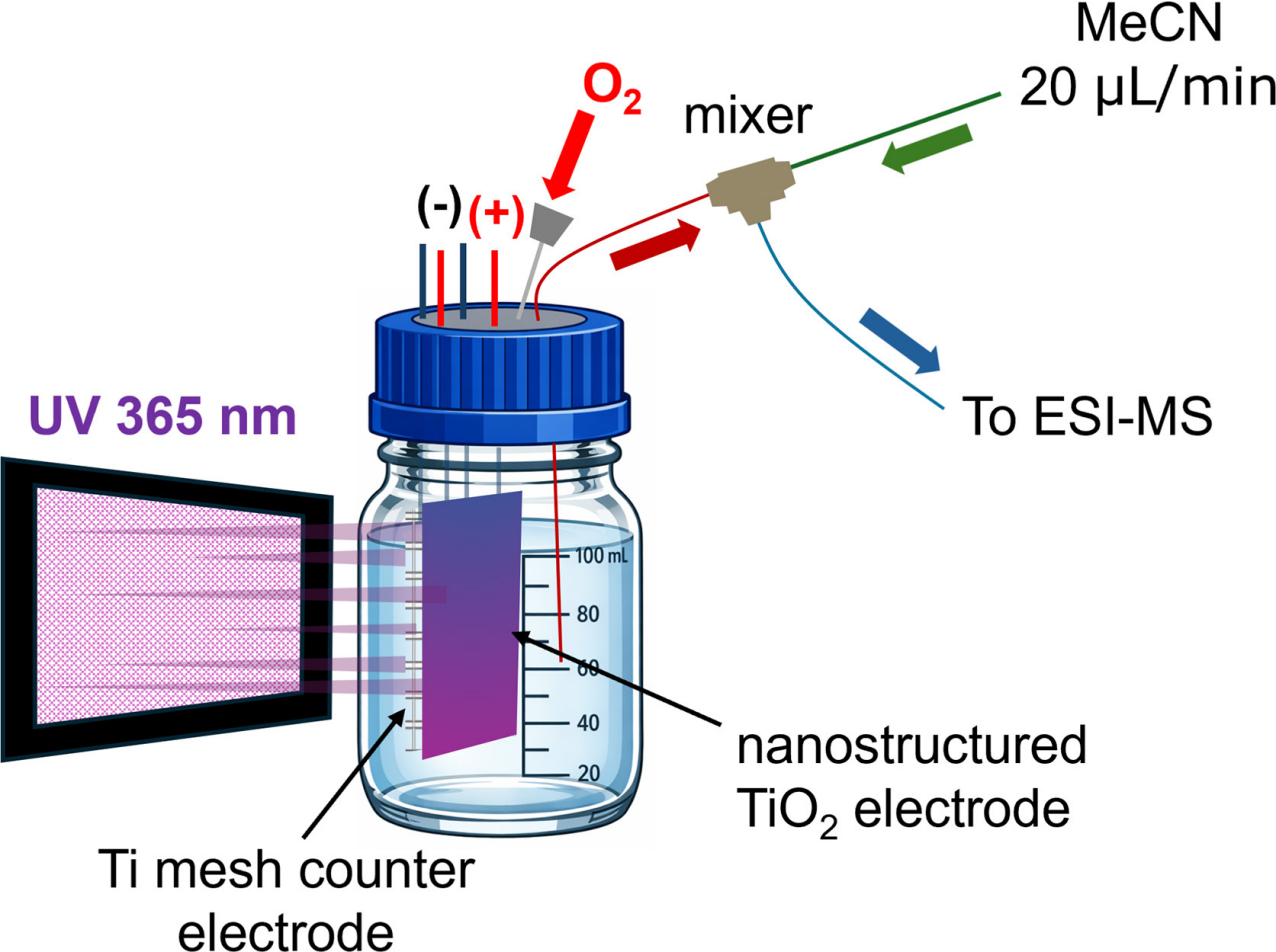
Experimental set up for real‐time photoelectrocatalytic monitoring by PSI‐MS. The reactor consists of a nanostructured TiO_2_ electrode and a Ti mesh counter electrode irradiated with 365‐nm UV light. Oxygen is supplied to the reaction flask at 5 psi. The aqueous reaction mixture was combined with acetonitrile (MeCN, 20 μL min^−1^) prior to introduction to the ESI‐MS.

### 2.2 Rhodamine B Analysis

Rhodamine B (1 μM) degradation as a function of time was investigated under three conditions: (i) in DI water with TiO_2_ under PEC conditions (2.0 V, 365‐nm UV light; measured emission maximum ∼373 nm Figure S3); (ii) in DI water with added HCl; and (iii) in DI water under UV irradiation in the absence of catalyst (photolysis control). For the HCl experiment, the HCl solution was prepared by a 1:1 (v/v) dilution of concentrated HCl (36.5–38.0 wt%, ACS grade) with DI water. Approximately 30 μL was injected into 100‐mL reaction solution after stabilization of the PSI‐MS signal and prior to UV irradiation and bias application, resulting in a final chloride concentration of approximately 1.8 mM and an estimated pH of approximately 2.7. Experiment durations ranged from 120 to 350 min depending on the condition investigated. Data were processed by binning into 60‐s intervals to improve signal‐to‐noise ratios. Binning was performed by averaging all data points within a 60 s interval. Data were also normalized to the total ion current (TIC).

Real‐time monitoring was performed using a Waters Synapt G2‐Si HDMS quadrupole time‐of‐flight mass spectrometer (QTOF‐MS, Waters Corporation, Milford, MA, United States) equipped with an electrospray ionization (ESI) source. Positive ion mode was used to monitor the parent ion and major transformation products, whereas negative‐ion mode was used to identify additional intermediates. Instrument parameters were optimized using *OptiMS* Python‐based software [33]. The system was operated with a capillary voltage of 3.0 kV, cone voltage of 40 V, 80‐V offset, source temperature of 100°C, desolvation temperature of 300°C, 50 L h^−1^ cone gas, 400 L h^−1^ desolvation, and 2.8‐bar nebulizer for positive mode. In negative mode, the MS was operated with a capillary voltage of 2.5 kV, cone voltage of 40 V, 0‐V offset, source temperature of 100°C, and desolvation temperature of 300°C, 50 L h^−1^ cone gas, 400 L h^−1^ desolvation gas, and 2.8‐bar nebulizer pressure.

## 3 Results and Discussion

The pressurized sample infusion (PSI) configuration using a media bottle with a rubber septum enabled stable, continuous monitoring of Rhodamine B, and its transformation products in real time. The PSI setup required an initial equilibration period of approximately 10 min, after which a stable ion signal was maintained for the duration of the experiment. The continuous acquisition allowed detection of the parent ion ([C_28_H_31_N_2_O_3_]^+^ = [M]^+^, *m/z* 443.3; Figure S4), transformation products such as an oxidized product [M + O]^+^ at *m/z* 459.3, and additional lower‐intensity transformation products, including ions at *m/z* 165.0 and 137.0 (Figure 2), allowing both major and minor reaction‐related species to be followed over time. The structures shown in Figure 2 are proposed based on the observed masses and their relationship to the Rhodamine B structure and are consistent with previously reported Rhodamine B degradation products, including phthalic acid and hydroxybenzoic acid species [30]. The parent ion and transformation products exhibited time‐dependent intensity changes, enabling direct observation of reaction progress (Figure 3). Approximately 120 data points were obtained after averaging the data into 60‐s time bins, providing higher time resolution than off‐line monitoring, which is limited to discrete sampling [22]. The degradation of Rhodamine B in DI water was performed in triplicate to assess reproducibility of the PSI setup for *m/z* 443.2 (Figure 4). The HCl and photolysis experiments were included as qualitative comparisons to demonstrate the ability of PSI‐ESI‐MS to distinguish condition‐dependent reaction behavior and were therefore performed as single representative experiments. This increased time resolution enables transient species to be resolved based on their time‐dependent behavior, facilitating differentiation between evolving reaction products and persistent background signals. Additional degradation and control experiments are provided in the Supporting Information (Figures S5–S8). The observed differences in degradation behavior reflect variations in catalyst preparation and reactor configuration, with the SI data processed using 30 s bins, highlighting the utility of PSI‐ESI‐MS for comparing and monitoring heterogeneous photocatalytic systems across different experimental designs.

**FIGURE 2:**
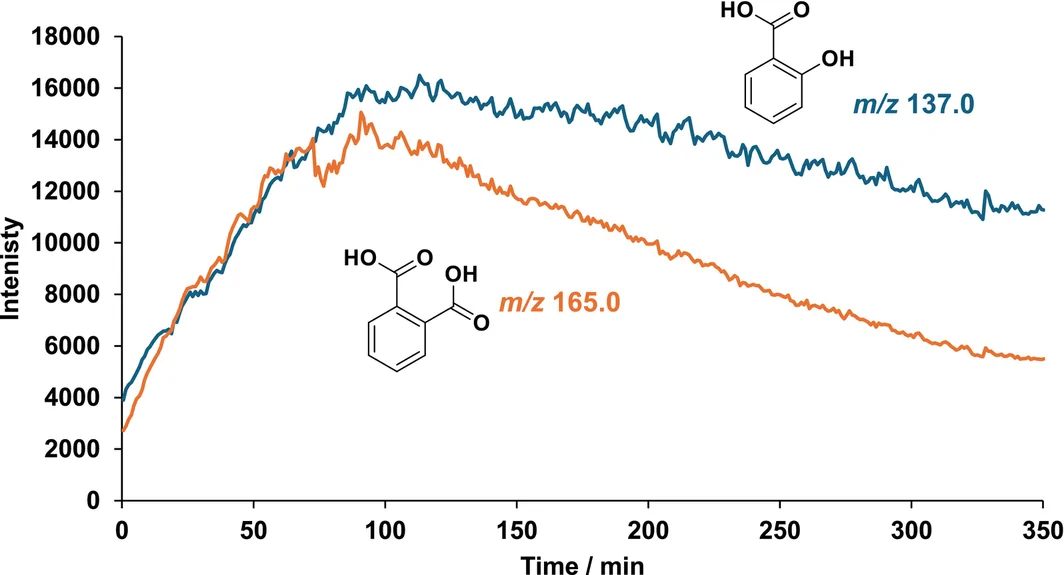
Low‐intensity intermediate ions with proposed structural assignments, showing a distinct rise‐and‐fall over time for species found at *m/z* 165.0 (bottom, orange) and *m/z* 137.0 (top, blue) in negative‐ion mode.

**FIGURE 3:**
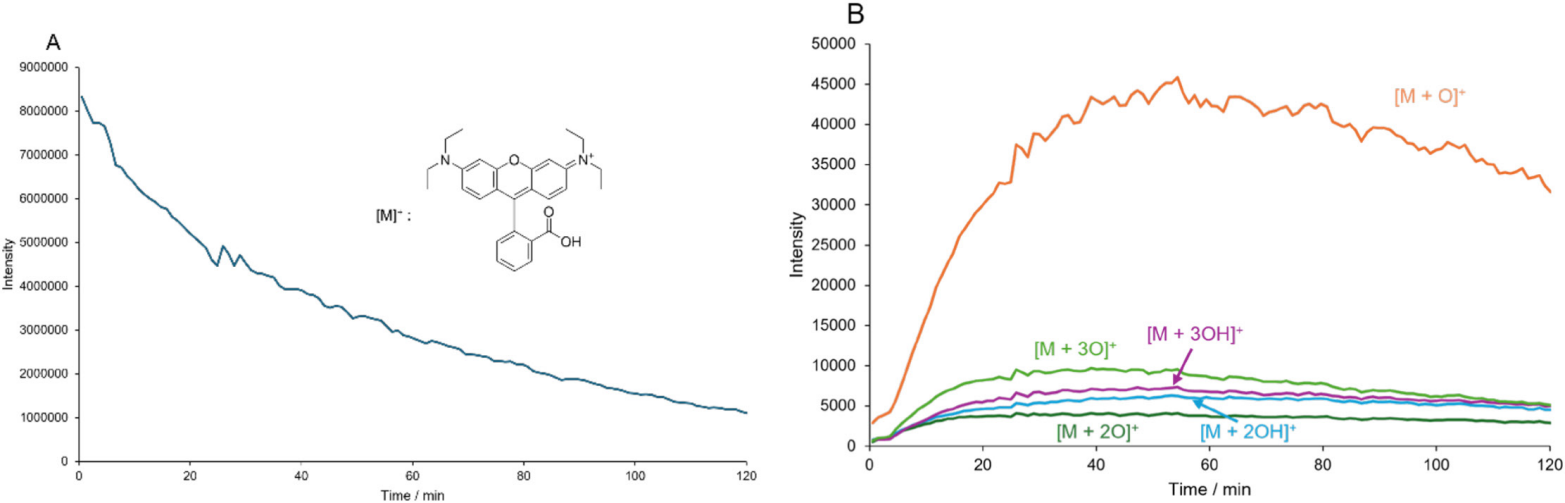
PSI‐ESI‐MS degradation profiles of (A) *m/z* 443.3 (parent ion, Rhodamine B) and (B) oxidized transformation products with TiO_2_ nanotube catalyst, 365‐nm UV light, and 2.0‐V applied bias in water over 120 min.

**FIGURE 4:**
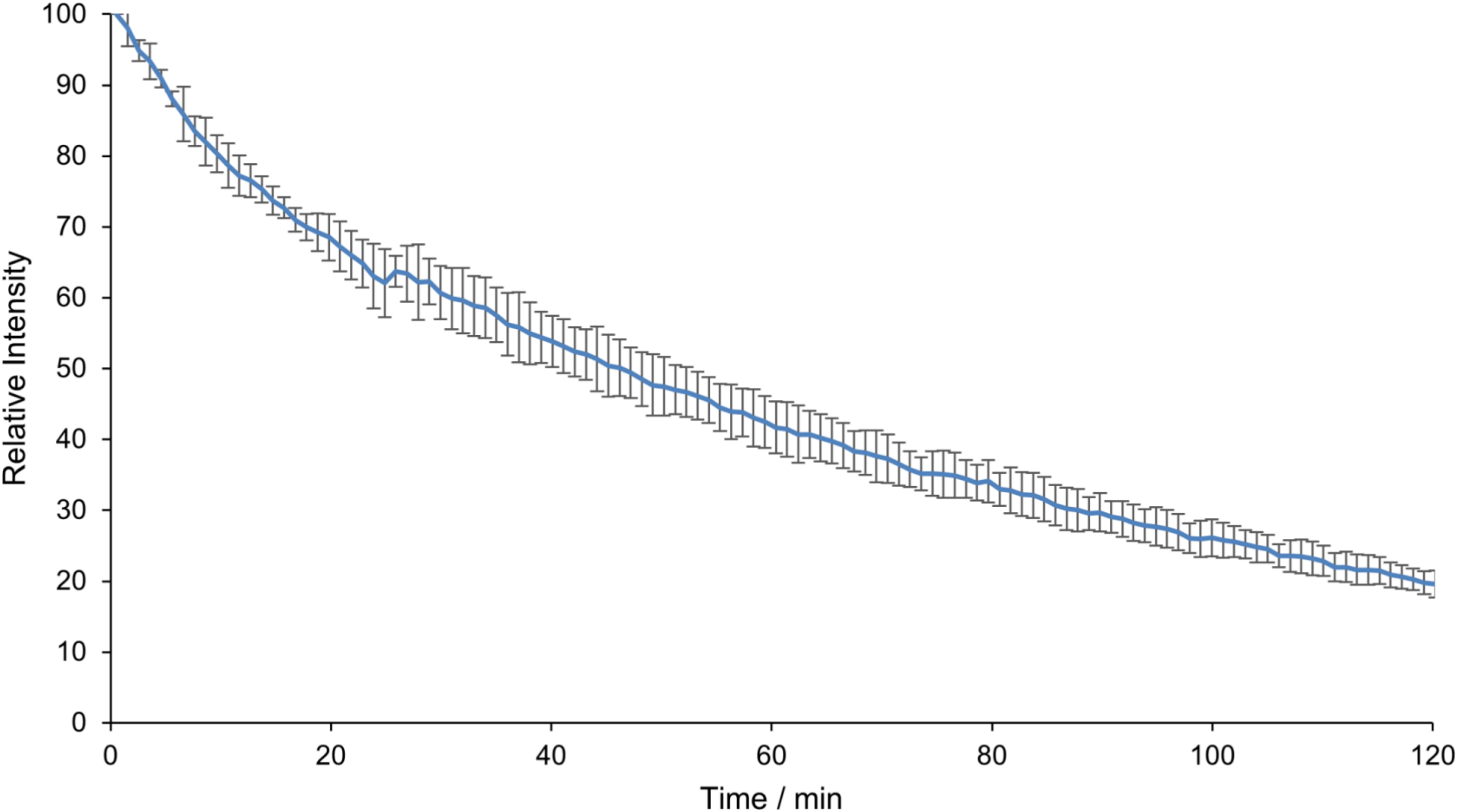
PSI‐ESI‐MS degradation profile of *m/z* 443.3 acquired in triplicates with TiO_2_ nanotube catalyst, 365‐nm UV light, and 2.0‐V applied bias in water over 120 min.

To evaluate the ability of PSI‐ESI‐MS to distinguish condition‐dependent reaction behavior, Rhodamine B was examined under photolysis, PEC, and chloride‐containing reaction conditions. A measurable decrease in the parent ion signal was also observed under UV irradiation in the absence of a catalyst, indicating that direct photolysis contributes to Rhodamine B degradation (Figure S9) [34]. Under otherwise comparable UV irradiation conditions, greater parent ion depletion and the appearance of additional intermediate species were observed in the representative experiment containing the TiO_2_ nanotube catalyst compared with the photolysis control. In the presence of HCl, a distinct change in reaction behavior was observed when compared to DI water conditions (Figure S10). Upon addition of HCl to the reaction system, the parent ion (*m/z* 443.3) decreased substantially within approximately 5–10 min, accompanied by the appearance of new ions at *m/z* 477.2 [M‐H + Cl]^+^, 511.2 [M‐2H + 2Cl]^+^, and 545.1 [M‐3H + 3Cl]^+^, corresponding to progressively chlorinated Rhodamine B derivatives. The experimental isotope patterns of the ions at *m/z* 477.2, 511.2, and 545.1 agreed with theoretical chlorine‐containing isotope distributions, supporting their assignment as monochlorinated, dichlorinated, and trichlorinated Rhodamine B derivatives, respectively (Figure S11). Under otherwise identical conditions in DI water, these chlorinated products were not observed at comparable intensity. Whereas UV‐Vis measurements showed rapid decolouration of Rhodamine B under chloride‐containing conditions, real‐time ESI‐MS demonstrated that the decrease in parent ion signal coincided with the formation of chlorinated transformation products rather than complete mineralization. The formation of chlorinated transformation products is consistent with previous reports of TiO_2_‐based photoelectrocatalytic systems, where halide ions are oxidized to reactive halide species that subsequently react with organic substrates to yield halogenated products across a broad range of compounds [35, 36, 37, 38]. These comparison experiments were included to demonstrate the ability of the modified PSI‐ESI‐MS setup to resolve condition‐dependent changes in degradation behavior and product formation in real time, rather than to establish the complete degradation mechanism of Rhodamine B under each condition.

The media‐bottle PSI‐ESI‐MS configuration reduces experimental cost and complexity while expanding PSI applicability to systems that use (relatively) large plate‐based catalysts. Scaling conventional Schlenk‐based PSI setups to larger volumes presents practical limitations, particularly due to the difficulty in sourcing appropriately sized septa and the need for custom glassware fabrication. In contrast, commercially available media bottles equipped with septum‐compatible screw caps provide a simple and low‐cost alternative. The rubber septum allows for a tight fit between the screw‐on lid and the glass media bottle, enabling pressurization within the reaction vessel, while still maintaining a seal during insertion of both the gas injection needle and PEEK tubing. Repeated needle and PEEK tubing insertion into the septum can lead to gradual degradation of the septum, potentially resulting in minor leaks over extended experiments. However, when the rubber septum was replaced periodically, no observable impact on signal stability or overall system performance was detected under the conditions used in this study, indicating that sufficient pressure was maintained throughout the experiments.

Despite this increased flexibility, the setup still retains practical limitations, particularly for very large or highly irregular catalyst geometries and systems requiring higher pressures, highly viscous media, or complex multiphase flow, which may require more robust sealing and pumping strategies beyond the current configuration. A possible approach to accommodate larger systems is the use of an enclosed, pressurizable vessel equipped with a rubber septum that allows insertion of PEEK tubing while maintaining system integrity. An additional challenge arises from efficient mixing of the aqueous phase with acetonitrile within the static mixer, which is limited by diffusion under laminar flow conditions [39]. This issue was mitigated by sonication of the static mixer and thorough rinsing of PEEK tubing with acetonitrile after each experiment, which improved subsequent experimental mixing and flow performance. Maintaining consistent pressure was achieved by periodically replacing the rubber septum, as repeated puncturing by needles and PEEK tubing led to material degradation and compromised sealing over time. Despite these limitations, the system maintained stable flow and signal over the duration of typical experiments (1–3 h), indicating that these mitigation strategies were sufficient for reproducible operation (Figure 4). The dead volume within the PEEK tubing may introduce a slight time offset in measured kinetic data. This delay depends on parameters including the tubing length and internal diameter, applied pressure, and solution viscosity, and the corresponding flow rate can be estimated using the Hagen–Poiseuille relationship [2, 21]. Previous PSI studies have reported transport delays on the order of 5–20 s [21]. For the relatively slow reactions examined here, this delay is small relative to the overall experimental timescale (≥ 120 min). However, for quantitative kinetic measurements, particularly for faster reactions, the transport delay should be determined or calibrated for the specific experimental configuration.

## 4 Conclusion

A modified PSI‐ESI‐MS configuration was successfully adapted for monitoring heterogeneous plate‐based photocatalytic systems using an enclosed, septum‐fitted media bottle. Stable flow and signals were maintained over 1‐ to 3‐h experiments, allowing for continuous real‐time monitoring of Rhodamine B degradation and the formation of oxidation products under photoelectrocatalytic conditions. This enabled clear differentiation between true reaction transformation products, which showed rise‐and‐fall behavior, and persistent background contaminants, which remained relatively constant over time. This distinction is a major advantage of using the PSI‐ESI‐MS system, as conventional off‐line monitoring makes it more difficult to separate background noise from true reaction products. Although practical limitations remain for highly viscous and complex multiphase systems, this configuration expands the accessible range of PSI‐ESI‐MS and provides a simple, adaptable platform for time‐resolved reaction monitoring in plate‐based and immobilized heterogeneous catalytic systems.

## Funding

This work was supported by the Natural Sciences and Engineering Research Council of Canada (NFRFE‐2023‐00213), Mitacs, the Michael Smith Health Research BC Foundation, and the Ryane Innovative Research Labs Inc.

## Supporting information




**Figure S1:** Vertically arranged extracted‐ion traces for *m/z* 459.2 (top) and *m/z* 282.3 (bottom), displayed separately for comparison. The *m/z* 459.2 exhibits transient rise‐and‐fall behavior consistent with an intermediate, whereas *m/z* 282.3 exhibits no distinct rise or fall behavior, indicating a signal consistent with background contamination.
**Figure S2:** Construction and assembly of the PSI‐PEC reactor. (A) PEEK tubing, syringe, and mixer used for sample transfer. (B) Reactor positioned adjacent to the UV‐LED source. (C) Ti mesh counter electrode from the modified GL45 cap. (D) Electrical connections to the reactor electrodes. (E) O_2_ inlet for vessel pressurization. (F) PEEK tubing inserted into the reactor for sample transfer. (G) TiO_2_ nanotube working electrode suspended from the modified GL45 cap.
**Figure S3:** UV‐Vis spectra of commercial 365 nm UV‐LEDs using an Ocean Optics FLAME‐T portable spectrometer equipped with a #9–1200 Line Holographic VIS grating placed 30 cm from the fiber‐optic cable. Spectrometer settings were a 1000 ms integration time, 10 scans averaged, and a boxcar width of 0. The green trace represents the 50 W flood UV‐LED, and the purple trace represents the 12 W tri‐LED.
**Figure S4:** Spectrum of the parent ion of Rhodamine B at m/z 443.2, with theoretical overlay, when run under positive mode with a concentration of 1.0 μM in DI water, mixed in the static mixer with acetonitrile.
**Figure S5:** Degradation trace of RhB (purple, m/z 443.3) and control (green, no catalyst), using 1 μM RhB, 365 nm UV‐LED, 2.0 V, processed using 30 s bins of 1 s scans, normalized to TIC before relative to starting peak of RhB parent peak (m/z 443.3).
**Figure S6:** Degradation trace of [RhB + O] (m/z 459.2) and control (green, no catalyst), using 1 μM RhB, 365 nm UV‐LED, 2.0 V, processed using 30 sbins of 1 s scans, normalized to TIC before relative to starting peak of RhB parent peak (m/z 443.3).
**Figure S7:** Degradation trace of [RhB—CH_2_CH_2_] (m/z 415.2) and control (green, no catalyst), using 1 μM RhB, 365 nm UV‐LED, 2.0 V, processed using 30 s bins of 1 s scans, normalized to TIC before relative to starting peak of RhB parent peak (m/z 443.3).
**Figure S8:** Triplicate Rhodamine B degradation experiments, with individual runs shown in light green, light blue, and dark green, and the mean shown in purple with standard deviation.
**Figure S9:** PSI‐ESI‐MS degradation profiles of the Rhodamine B parent ion (m/z 443.3) under PEC conditions (red, TiO_2_ nanotube catalyst, 2.0 V bias, 365 nm UV‐LED) and UV irradiation only (blue, no catalyst or applied bias) in DI water over 120 min.
**Figure S10:** PSI‐ESI‐MS degradation profiels of Rhodamine B under PEC conditions with HCl added (Blue) and without HCl (Orange), using a TiO_2_ nanotube catalyst, Ti mesh counter electrode, 2.0 V, and 365 nm UV‐LED.
**Figure S11:** Experimental (black traces) and theoretical (pink bars) isotope patterns of chlorinated Rhodamine B transformation products observed during PSI‐ESI‐MS monitoring under PEC conditions in the presence of HCl. (A) Monochlorinated Rhodamine B at *m/z* 477.2, (B) dichlorinated Rhodamine B at *m/z* 511.2, and (C) trichlorinated Rhodamine B at *m/z* 545.1.

**Data S1:** Supporting Information.

## Data Availability

The data that support the findings of this study are available in the Supporting Information.
