# Supplementary material for: Adapting Pressurized Sample Infusion ESI‐MS for Real‐Time Monitoring of Heterogeneous Photoelectrocatalytic Reactions
Source: J Mass Spectrom. 2026 Nov 1;61(11):e70129. doi: 10.1002/jms.70129 (PMC13725125; doi:10.1002/jms.70129)
Supplement: Supplementary file 1 [file JMS-61-e70129-s001.docx]

**Supporting Information**

**Adapting Pressurized Sample Infusion ESI-MS for Real-time Monitoring of Heterogeneous Photoelectrocatalytic Reactions**

Bobby A. Ryane, Bragi Zhang, Maria E. Muraro, Hannah A. Trieu, Heather L. Buckley and J. Scott McIndoe

Theoretical isotope distributions shown in Figures S4 and S11 were generated using a Python script.[^40^](https://www.zotero.org/google-docs/?rcREfP) Raw data used to generate figures available in the supporting information as PSI data.zip.

**Figure S1.** Vertically arranged extracted-ion traces for *m/z* 459.2 (top) and *m/z* 282.3 (bottom), displayed separately for comparison. The *m/z* 459.2 exhibits transient rise-and-fall behaviour consistent with an intermediate, whereas *m/z* 282.3 exhibits no distinct rise or fall behavior, indicating a signal consistent with background contamination.

**Figure S2**. Construction and assembly of the PSI-PEC reactor. (A) PEEK tubing, syringe, and mixer used for sample transfer. (B) Reactor positioned adjacent to the UV-LED source. (C) Ti mesh counter electrode from the modified GL45 cap. (D) Electrical connections to the reactor electrodes. (E) O_2_ inlet for vessel pressurization. (F) PEEK tubing inserted into the reactor for sample transfer. (G) TiO_2_ nanotube working electrode suspended from the modified GL45 cap

**Figure S3.** UV-Vis spectra of commercial 365 nm UV-LEDs using an Ocean Optics FLAME-T portable spectrometer equipped with a #9 - 1200 Line Holographic VIS grating placed 30 cm from the fiber-optic cable. Spectrometer settings were a 1000 ms integration time, 10 scans averaged, and a boxcar width of 0. The green trace represents the 50 W flood UV-LED, and the purple trace represents the 12 W tri-LED.

**Figure S4.** Spectrum of the parent ion of Rhodamine B at m/z 443.2, with theoretical overlay, when run under positive mode with a concentration of 1.0 μM in DI water, mixed in the static mixer with acetonitrile.

**Figure S5.** Degradation trace of RhB (purple, m/z 443.3) and control (green, no catalyst), using 1 µM RhB, 365 nm UV-LED, 2.0 V, processed using 30 s bins of 1 s scans, normalized to TIC before relative to starting peak of RhB parent peak (m/z 443.3).

**Figure S6.** Degradation trace of [RhB + O] (m/z 459.2) and control (green, no catalyst), using 1 µM RhB, 365 nm UV-LED, 2.0 V, processed using 30 sbins of 1 s scans, normalized to TIC before relative to starting peak of RhB parent peak (m/z 443.3).

**Figure S7.** Degradation trace of [RhB - CH_2_CH_2_] (m/z 415.2) and control (green, no catalyst), using 1 µM RhB, 365 nm UV-LED, 2.0 V, processed using 30 s bins of 1 s scans, normalized to TIC before relative to starting peak of RhB parent peak (m/z 443.3).

**Figure S8.** Triplicate Rhodamine B degradation experiments, with individual runs shown in light green, light blue, and dark green, and the mean shown in purple with standard deviation.

**Figure S9.** PSI-ESI-MS degradation profiles of the Rhodamine B parent ion (m/z 443.3) under PEC conditions (red, TiO_2_ nanotube catalyst, 2.0 V bias, 365 nm UV-LED) and UV irradiation only (blue, no catalyst or applied bias) in DI water over 120 min.

**Figure S10.** PSI-ESI-MS degradation profiels of Rhodamine B under PEC conditions with HCl added (Blue) and without HCl (Orange), using a TiO_2_ nanotube catalyst, Ti mesh counter electrode, 2.0 V, and 365 nm UV-LED.

**Figure S11.** Experimental (black traces) and theoretical (pink bars) isotope patterns of chlorinated Rhodamine B transformation products observed during PSI-ESI-MS monitoring under PEC conditions in the presence of HCl. (A) Monochlorinated Rhodamine B at *m/z* 477.2, (B) dichlorinated Rhodamine B at *m/z* 511.2, and (C) trichlorinated Rhodamine B at *m/z* 545.1.
